# Vitamin D and Hyperkinetic Movement Disorders: A Systematic Review

**DOI:** 10.5334/tohm.74

**Published:** 2020-08-25

**Authors:** Carl N. Homann, Gerd Ivanic, Barbara Homann, Tadeja Urbanic Purkart

**Affiliations:** 1 Department of Neurology, Medical University Graz, Graz, AT; 2 St. Elizabeth University of Health and Social Work, Bratislava, SK; 3 Institute for Orthopedic and Cardiological Rehabilitation, Privatklinik Ragnitz, Graz, AT

**Keywords:** Vitamin D, movement disorders, hyperkinetic movements, fractures, bone structure

## Abstract

**Background:**

The importance of vitamin D deficiency in Parkinson’s disease, its negative influence on bone health, and even disease pathogenesis has been studied intensively. However, despite its possible severe impact on health and quality of life, there is not a sufficient understanding of its role in other movement disorders. This systematic review aims at providing an overview of the prevalence of vitamin D deficiency, bone metabolism alterations, and fractures in each of the most common hyperkinetic movement disorders (HKMDs).

**Methods:**

The study search was conducted through PubMed with keywords or Medical Related Subjects (MeSH) of common HKMDs linked with the terms of vitamin D, osteoporosis, injuries, and fractures.

**Results:**

Out of 1585 studies screened 40 were included in our review. They show that there is evidence that several HKMDs, including Huntington disease, Restless Legs Syndrome, and tremor, are associated with low vitamin D serum levels in up to 83% and 89% of patients. Reduced bone mineral density associated with vitamin D insufficiency was described in Huntington disease.

**Discussion:**

Our survey suggests that vitamin D deficiency, bone structure changes, and fractures are important but yet under-investigated issues in HKMDs. HKMDs-patients, particularly with a history of previous falls, should have their vitamin D-levels tested and supplemented where appropriate.

**Highlights:**

Contrary to Parkinson’s disease, vitamin D deficiency, and bone abnormalities are under-investigated in hyperkinetic movement disorders (HKMDs). Several HKMDs, including essential tremor, RLS, and Huntington disease, are associated with vitamin D deficiency in up to 89%, the latter also with reduced bone mineral density. Testing and where appropriate supplementation is recommended.

## Introduction

For a long time, vitamin D has been described as being vital for our body as it regulates calcium homeostasis and maintains bone integrity. However, in the last ten years, research has delineated the important role it plays for various other organ systems, especially the brain. Vitamin D has been associated with many distinct neurological functions, particularly neuroprotection. Insufficient levels have consequently been linked to an increased likelihood of developing neurological diseases such as movement disorders, neuromuscular disorders, multiple sclerosis, and dementia [1].

Substantial progress within the last decade has been achieved in understanding the importance of vitamin D in PD. This helped to reveal that in patients suffering from PD, vitamin D insufficiency appears to be common [2], not only leading to poor bone health [3] but also being pointed out as a possible factor for disease development and progression. The effects thereof are considered relevant for poor quality of health and augmented health care costs [4]. Given the pathophysiological commonalities, one might assume that similar findings can be expected in HKMDs. However, to date, there has been a relative scarcity of studies investigating vitamin D and related issues in HKMDs. This study, therefore, is aimed at providing a systematic overview of the current state of data on the prevalence of vitamin D deficiency, possible causes and consequences, and the value of supplementation.

Why now would it be especially important to investigate vitamin D in HKMDs? On a scientific level, research into the role of vitamin D might help us further deepen our understanding of the pathomechanism of neurodegenerative diseases, especially but not limited to basal ganglia disorders. On a practical level, we hope to find in vitamin D a remedy that might be potentially effective in mitigating disease development and progression, and in reducing some of the disabling motor symptoms like balance problems and some of the non-motor symptoms like mental and emotional dysfunctions so commonly associated with movement disorders [5]. However, vitamin D also deserves our attention because it might prevent or reduce some of the secondary conditions like fractures due to disease-related falls. Injuries and fractures in HKMDs can have substantial consequences, potentially contributing to reduced activity levels [6], poor quality of life [6,7], and institutionalization [4,8]. Given the still limited level of knowledge, this study should help us to direct our focus to where we need to do more research and hopefully, where already now we can draw a relevant conclusion on how to advise our patients better.

## Methods

The study search was conducted through PubMed for all articles in English, French, and German until March 1, 2020. As part of a standard search protocol, the following keywords or Medical Related Subjects (MeSH) were used to specify the literature search: (“vitamin D” or “ergocalciferols”); together with (“Restless leg syndrome” or “RLS”); (“Chorea” or “Huntington”); “Dystonia”, (“Tremor” or “Essential Tremor”); “myoclonus”; “athetosis”; (“Tic” or “Tourette”), “stereotypies”, and “ballism”.

Also, a link was made to each of these medical condition with (“bone mineralization” or “bone mineral density”), (“osteoporosis” or “osteopenia”), (“injuries” or “fractures”) as well as “pathological fractures”. In addition, the bibliographies of the identified publications were reviewed for further appropriate studies. Dual publications were singled out for exclusion by comparison of author name, intervention, publication date, sample sizes, and results.

## Results

### Article selection

**Figure 1:**
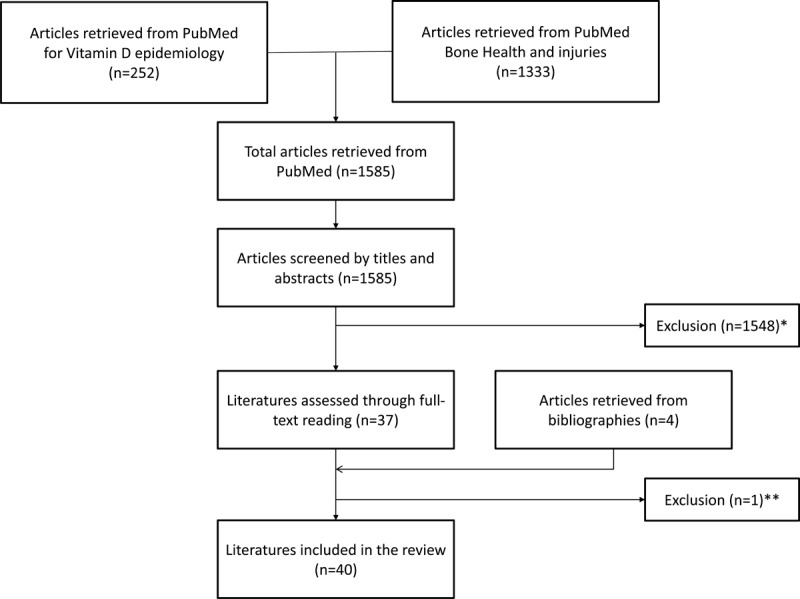
**Flow Diagram for Identification of Relevant Studies.** Reasons for exclusion: *studies not related to search topic, non-human (i.e. animal) studies, study language other than English, German, or French, or no new data presented (editorials, reviews etc.). **duplicate publication.

Through the above described systematic search strategy, we retrieved 252 studies combining HKMDs with vitamin D and 1333 studies linking HKMDs with bone health and fractures. When screened by titles and abstracts, we excluded 1548 studies not meeting the inclusion criteria. Through full-text reading, we removed one duplicate publication but added four retrieved from bibliographies, leaving us with 40 scientific articles to use for this overview. (Figure 1).

### Huntington disease

Vitamin D deficiency appears to be common among Huntington disease patients, although large-scale population-based studies have yet to be performed. Chel et al. observed in a study on 28 patients with manifest disease average serum-25 (OH) D-levels of 33 nmol/L [9]. In total 89% of the subjects had a vitamin D insufficiency (25 (OH) <50 nmol/L) [9].

There is some evidence that there is a disease-specific alteration in bone metabolism in patients with Huntington disease. Costa de Miranda et al. discovered in a study on 21 patients and 29 age- and sex-matched healthy controls that bone mineral density and T-scores were lower in the Huntington group [10]. Goodman et al. detected in 25 high-risk individuals, i.e., already prior to the outbreak of Huntington disease, significantly lower bone mineral density (Z-scores) compared to 25 control subjects [11]. There was, however, in this small sample, no statiscally relevant correlation with vitamin D levels nor with other active protein structures like testosterone, cortisol, and leptin. Thus, the authors considered early gene-induced processes to be the most probable cause. Data from two preliminary reports suggest that osteoporosis could be part of the Huntington phenotype [12,13]. It has also been shown that the severity of osteoporosis correlates with the number of CAG repeats [13], which is the most frequently evaluated marker of disease severity.

Fracture risk is high in patients with Huntington disease [6]. Authors see this in relation to the elevated propensity for falls [14]. However, there is no data on whether the relative risk of injuries per fall is also elevated. These are relevant issues for patients with Huntington’s, because fractures, falls and as a consequence impairment of independent ambulation often results in the loss of autonomy and transfer to nursing homes [8]. To our knowledge, there is unfortunately no investigation on the connection of vitamin D deficiency and fractures and also nothing on pathological fractures.

Possible disease-specific effects of vitamin D deficiency in Huntington disease have been discussed. The study by Chel et al. described above saw a positive inverse association between serum 25 (OH) D levels and functional impairment [9]. However, the patient cohort was small and the nature of the study design does not allow for causal assumptions. Up to now, there are no substitution studies in patients with Huntington’s disease. In our opinion, investigations of disease-specific or general effects of vitamin D administration, be it substitutional or prophylactic, should be of great interest.

### Restless Legs Syndrome

Only a few investigations looked at vitamin D status in RLS patients. Wali et al. [15] conducted a population-based case-control study to assess the association between RLS and vitamin D levels. In the data of his 78 patients with RLS and 123 controls, he ascertained that the risk for the development of RLS was significantly higher in vitamin D deficient cases when compared to those who are vitamin D sufficient (OR 3.1, *P* < .002, 95% CI 1.51–6.38). Additionally, the mean serum 25(OH) vitamin D level was significantly lower in patients with RLS than in normal controls (12.65 ng/mL vs. or 26.12 ng/mL, *P* < .001). Accordingly the number of deficient cases was higher in the patient than in the control group (75.6% vs. 42.3%, *P* < .001). Their data also showed a relationship between increased RLS severity measured using the International RLS Study Group Scale (IRLSSG) and reduced serum vitamin D levels (IRLSSG scores in deficient vs. sufficient cases: 19.4 ± 5.9 vs. 14.5 ± 4.7, *P* < .002).

In 83% of the 36 patients with RLS, Balaban et al. noticed serum 25-OH vitamin D3 levels to be decreased [16]. In female patients with RLS, mean serum levels were significantly lower than in female control subjects (p = 0.001). However, lumbar bone density, as measured by T-score was higher in comparison to controls, and thus fewer RLS patients than healthy control subjects had osteopenia [16]. This was interpreted as a consequence of increased mobility in RLS patients caused by the urge to move as a means to eliminate unpleasant sensations.

Some disease-specific factors seem to influence bone metabolism. Patients with RLS seem to frequently have increased sympathetic activation and increased nocturnal cortisol excretion in the urine compared to controls [17]. Both factors exert a negative influence on bone metabolism but have a positive effect on the function of dopaminergic neurons in the brain. It is also known that physiologically a reduced cortisol and dopamine release take place during night-time hours.

In a study by Cikrikcioglu et al. including 96 RLS patients and 97 healthy controls, patients with RLS had significantly lower levels of serum calcium, bone remodeling marker CTX (telopeptide of type I collagen) and sclerostin. These findings were associated with longer duration of illness and more severe RLS symptoms [18].

Increased fracture risk after falls, which seem to be frequent in RLS patients [19], has been described by Kuzinar et al. [19]. Unfortunately, he did not measure vitamin D levels, nor did he do any bone density examinations.

Vitamin D is also said to have disease-specific effects in RLS. For example, Balaban et al. reported on a correlation between low vitamin D values with increased disease severity [16]. A small open-label study by Wali et al. examined the effect of vitamin D supplementation. They showed in 12 vitamin D-deficient RLS patients after administration of vitamin D either as an oral dose of 28,000 IU per week or a parenteral dose of 200,000 IU i.m. per month that the vitamin D3 level could be successfully corrected to >50 nmol/l and that then a significant improvement in the severity of RLS symptoms occurred [20]. However, contrary to this, a recent 12-week double-blind placebo-controlled study on 22 completed cases by the same authors, established that vitamin D supplements at a dose of 50,000 IU caplets per os administered weekly were not effective in improving RLS symptoms [21]. The authors, acknowledging the limitations of this recent study, suggested that future investigations should comprise a longer study period, adequate sample size, and recruitment of patients with severer forms of RLS and most important with vitamin D deficits. In a more recent project, Tutuncu et al., taking into consideration this latest advice, examined the effect of supplementation on vitamin D deficient RLS patients [22]. In this prospective self-controlled case study, they treated 19 RLS-patients with 50,000 units per week for two months. vitamin D levels increased from 13.2 ± 4.0 to 42.8 ± 9.6 ng/mL while total scores of the International RLS Study Group scale (IRLSS) improved from 24.9 ± 5.1 to 21.1 ± 2.9 points (p < 0.001). Selected subscores also improved including symptom severity (p < 0.001), impact on sleep (p < 0.001), symptom measures (p = 0.002), and disease impact measures (p < 0.001). These findings differ from Wali’s 2019 results [21] but are broadly consistent with his earlier study [20]. Tutuncu explained these discrepancies by the fact that it seems to be important to target the treatment specifically to patients with Vitamin Deficiency, i.e., to those that really need it. However, as pointed out by Wali, large-scale studies would be of great value to settle those controvercies.

### Tic disorders

There are so far no population-based studies on vitamin D levels in tic disorders. However, a higher prevalence of vitamin D deficiency would be plausible, as several studies have indicated that when compared to typically developing children, those with developmental disabilities, including Tourette syndrome, participate in fewer outdoor activities [31,32]. Fewer hours spend outside relate to less skin sunshine exposure leading to reduced internal vitamin D production and lower levels [23,24].

There is still relatively little known about bone homeostasis in tic disorders. However, a recent publication by Rath et al. provides evidence for the alteration of bone metabolism in Tourette patients [25]. He assumes that vitamin D is involved but that this might primarily be drug-related. A common side effect of neuroleptics, the drug of first choice for the treatment of Tourette syndrome, is hyperprolactinemia. Up to 50% of people who take neuroleptics over a longer period may be affected [26]. There is convincing evidence that hyperprolactinemia leads to a reduction in bone mineral density through increased osteoclast and decreased osteoblast activation, as well as decreased vitamin D synthesis and calcium absorption [27]. An increase in prolactin levels appears to be particularly detrimental in young, still-growing patients who have not yet reached the maximal bone mass [27]. The study cited above describes two cases of pronounced hyperprolactinemia and decreased bone mineral density in Tourette patients [25]. Whether in tic disorders, vitamin D deficiency by itself, i.e., aside from drug side effects, also plays a significant role for bone mineralization, has not yet been investigated.

Concerning fall and fracture risk in patients with simple tics, there are only individual case reports. For complex tic disorders, there is somewhat more data available. Cheung et al. described a case of malignant Tourette syndrome, with recurrent falls and injuries, including skull fractures [7]. Fortunately, we now also have a recent large-scale systematic population-based investigation on fractures in patients suffering from Tourette syndrome. In this study, Lu et al. [28] uncovered that the cohort of 1258 Tourette patients had a 1.27-fold higher incidence of fractures than did the age and sex-matched comparison cohort (190.37 vs. 149.94 per 10,000 person-years). This included predominantly fractures of the skull, neck, and spine. Unfortunately, neither the systematic study nor the case report investigated a correlation of fracture risk and bone mineralization abnormalities or vitamin D status.

Regarding specific effects of vitamin D on tic disorders, we only found a small study by Gemawat et al. His data from 34 children with various forms of tics disorders suggests that after vitamin D supplementation there is an improvement reflected by a reduction in frequency, duration, and intensity of tic movements [29].

All these findings on tic disorders, however, have the limitation that they drew their data exclusively from children, and therefore, it is not known to what extent they can be extrapolated for the grown-up or elderly population. Thus, studies in adult are highly warranted.

### Essential tremor

In the literature search, we did not find any systematic studies regarding vitamin D status in essential tremor. There is only one case report that describes tremor as a probable, possibly even indicative symptom of vitamin D deficiency in children [27]. There are also no studies on bone health or fracture frequency for essential tremor.

Of interest are some recent genetic findings. There seems to be a link between the occurrence of essential tremor and vitamin D receptor polymorphism. In a case-control genetic association study on 239 sporadic essential tremor patients and 239 healthy controls, Sazci et al. analyzed the rs2228570 variant of the human vitamin D receptor gene [30]. The rationale behind this project was that genetic studies on vitamin D receptor polymorphisms had indicated its involvement in neurological disorders like PD [31]. In keeping with this, the expression level of vitamin D receptor mRNA had been identified as a potential blood biomarker for PD. Sazci investigated whether a similar association might also be present in essential tremor. He demonstrated for the first time that the rs2228570 variant of the vitamin D receptor gene is, in fact, associated with sporadic essential tremor overall and particularly in male patients. He, therefore, concluded that the rs2228570 variant should be considered to be a genetic risk factor for sporadic essential tremor. Whether and in as much this receptor polymorphism, however, is directly involved in the development of sporadic essential tremor he cautioned, remains to be elucidated [30].

### Dystonia

There are no studies examining vitamin D status in dystonia patients. A case report described dystonic choreoathetosis in a patient with familial idiopathic hypoparathyroidism [32]. The vitamin D levels, although presumably low, were not tested in the respective patient. Thus, no conclusion can be drawn on whether vitamin D deficiency would have been a confounding factor for the development of these dystonic symptoms.

We did not identify any systematic studies on bone mineralization in dystonia, but two reports on pathological fractures. Mohanty et al. reported on a case of mandibular angle and coronoid process fracture in a patient with orofacial dystonia [33]. They made the long-lasting mechanic traction through the dystonic facial musculature responsible for causing continuous gradual bone resorption to such an extent that the spontaneous fracture became inevitable. In another case report, McDade portrayed a man with L-dopa induced dystonia resulting in a metatarsal fracture [34]. Whether it resulted from the forces created by the inverted foot, or rather was a direct result of trauma from ambulating on it, he could not specify. In the absence of reported falls and by the pattern of the fracture, he assumed that this could be considered consistent with Jones fractures, which occur when a laterally directed force is applied to the forefoot of the plantar-flexed foot. It is unfortunate, and also possibly of relevance, that in none of the reports, we find references to the vitamin D status or the general bone metabolism.

Data on injuries for patients suffering from dystonia are scarce. A study on cervical dystonia patients suggests that these patients tend to fall more frequently [35], but it is not known whether the relative risk for fractures after those falls is also elevated. On the bases of the reported occurrence of pathological fractures and increased fall propensity, more scientific work on vitamin D status, injuries, and fall risk in all types of dystonia should be highly encouraged.

Vitamin D supplementation has been investigated by Habibi et al. in a double-blind placebo-controlled study on 120 patients with L-dopa-induced dyskinesia, including dystonia [36]. However, the global trial results where negative and no mention was provided on whether a sub-analysis was conducted on the group of dystonia patients. We believe that a large study including patients with all types of dystonia examining vitamin D supplementation would be of clinical benefit.

### Myoclonus

There are no systematic investigations on vitamin D status in myoclonus. We detected one historic case report on a 98-year-old woman with “myoclonia” and low serum concentration of vitamin D and calcium in which symptoms disappeared after supplementation [37]. Another case report describes vitamin D2 receptor alterations in a family of patients with myoclonus-dystonia [38].

There are no systematic investigations of bone mineralization in myoclonic patients, and there are also no publications on pathologic or traumatic fractures.

Only rudimentary data exist on gait and balance abnormalities [39,40] and none on falls and fracture risk. Further systematic studies on vitamin D status and bone structure abnormalities in patients with myoclonus are warranted.

### Other Hyperkinetic Movement Disorders

There are no studies on vitamin D status of the various other disorders with abnormal involuntary movements like stereotypies, akathisia, athetosis, and ballism. Neither are there investigations on bone mineralization, fractures or falls in these conditions.

## Discussion

This is the first systematic study that reviews the role of vitamin D and related issues in HKMDs. Considering that HKMDs are a group of common diseases and the effect of vitamin D deficiency can be severe, this is astonishing. In summary, we showed that many patients with HKMDs, although different in phenomenology and pathogenesis, have problems with vitamin D insufficiency and osteoporosis, but also with gait and balance. This often leads to falls and injuries. The role that vitamin D might play in this has hardly been investigated. Only some preliminary data exist on the disease-specific effect and supplementation. Altogether, the level of evidence for the investigated aspects differs widely, but in general, it is not very high. Some data of moderate quality is available for chorea and RLS, but only a few and incomplete investigations exist for essential tremor, dystonia, and myoclonus. No studies at all have been performed on stereotypies, akathisia, athetosis, and ballism.

### Vitamin D insufficiency and osteoporosis

There is evidence that several HKMDs, including Huntington disease, RLS, and tremor, are associated with low vitamin D serum levels. (Table 1) vitamin D deficiency, an essential factor for altered bone structure and increased fracture risk, was present in 89% of Chorea patients [9], and in 83% of RLS patients [16]. We found reduced bone mineral density associated with vitamin D insufficiency in Huntington disease but not in RLS patients [16]. For tic disorders, there is only one case report that describes a reduced bone mineral density in a patient with Tourette syndrome. However, the author attributed this pathology not to an altered vitamin D status but rather to neuroleptic-induced hyperprolactinemia [25].

**Table 1:** Characteristics of vitamin D prevalence studies in patients with hyperkinetic movement disorders.

Authors	Subjects (n)	Study design	Serum 25(OH)D, mean (SD)	Prevalence of deficiency	Correlated with	Compared to Co

Chel et al. [9]	HD inst.(28)	survey	33 ± 15 ng/mL	89%	walking disability	n.a.
Wali et al. [15]	RLS (78), Co (123)	case control study	12.65 ng/mL	76%		lower levels, higher deficiency prevalence
Balaban et al. [16]	f. RLS(36), f. Co (38)	cross sectional study	7.31 ± 4.63 ng/mL	83%	disease severity	lower levels, higher deficiency prevalence
Calarge et al. [27]	ET (1)	Case report		n.a.	disease severity	n.a.
Kato et at. [32]	Dys (1)	Case report		n.a.		n.a.
Gardiol et al. [37]	Myo (1)	Case report		n.a.		n.a.

HD: Huntington’s disease, RLS: Restless legs syndrom, ET: Essential Tremor, Dys: Dystonia, Myo: Myoclonus, Co: control subjects.

inst.: institutionalized; f.: female.

Compared to HKMDs, the evidence for lower vitamin D levels in hypokinetic movement disorders like PD is much more robust. Seven separate observational studies and a meta-analysis [2] have explored the relationship between vitamin D and PD and, except for one, persistently showed low serum 25 (OH) D levels in PD patients. In keeping with these findings, there are several publications, including a systematic review [3], that have demonstrated an altered bone structure in PD patients, with vitamin D deficiency likely to play a significant role. Reduced vitamin D levels in PD, are generally thought to be a consequence of the patients’ limited outdoor mobility due to hypokinesia and gait difficulties. Also, PD-specific digestive symptoms are blamed for reduced uptake [41]. We are not aware of any evidence for reduced sunlight exposure or gut abnormalities in HKMDs. Thus we were surprised to find the prevalence of vitamin D deficiency of some HKMDs like Huntington disease with 89% and RLS with 83% to match or even supersede those of PD with 57 to 71% [2]. This leaves room to explain vitamin D deficiency in movement disorders with its increased neurodegenerative risk [41]. In other words, vitamin D deficiency might not be the consequence of but rather a cause for the development of HKMDs. This chain of events has been assumed to be the case in other neurodegenerative diseases and other neurological conditions [1]. There are also suggestions based on experimental data that alterations in early life vitamin D status may influence the orderly development of parts of the basal ganglia and particularly of dopaminergic neurons [42].

Multiple studies detected reduced vitamin D levels and related high rates of osteoporosis, also in many other neurological conditions. They showed in patients with acute ischemic stroke the prevalence of vitamin D deficiency (<20,0 ng/mL) was 30–48,8% [43,44,45], and that in 77% levels were insufficient (<50 ng/mL) [46]. A deficiency was present in 23% of multiple sclerosis patients [47] and 45% of epilepsy patients [48]. Another 31% of epileptic patients had insufficient levels (20–29 ng/mL) [48]. Vitamin D insufficiency (<50 ng/mL) was also present in 81.5% of patients with diabetic neuropathy [49]. Evidence shows a reduced bone mass in stroke [46], multiple sclerosis [47], and epilepsy [48]. This was attributed to low sunlight exposure and reduced dietary intake, but in epilepsy, also to specific drug interactions [48].

### Fracture risk

An altered bone structure and increase fracture risk are particularly troublesome in patients with conditions that predispose them to frequent falls. Fall prevalence figures for chorea patients range from 60 to 79.2% [6,14], and for essential tremor patients from 50.7 to 58.7% [50]. For other HKMDs such as RLS [19] and tic disorders [7], there are but single reports of repeated falls. Falls in HKMDs can especially in the presence of vitamin D deficiency lead to an increased propensity of injuries and fractures as shown in Huntington disease, and Tourette syndrome, contributing to reduced activity levels [6], poor quality of life [6,7], and institutionalization [4,8]. As for epidemiological data on vitamin D deficiency or osteoporosis, here again, the data situation for PD is considerably better than for HKMDs. There are at least five investigations on fall-related injuries in PD patients. This research suggests that in PD, these injuries occur in up to 50% of falls [51] and that they constitute one of the top causes of increased health services utilization and overall costs [4]. Given the seemingly high prevalence and potentially grave consequences of falls and fractures in HKMDs patients, we find it astonishingly that so far, no comparative studies on this matter exist. As there is also no high-quality data on pathological fractures in HKMDs, this too could be a valuable future project.

Also, we had to notice a complete lack of studies investigating the influence of vitamin D deficiency on balance problems and falls, on the severity of injuries, and the healing process in the HKMD-population. This strongly contradicts the data situation for the healthy population, where there is an adequate amount of studies on this topic [52,53]. To pursue this further in HKMDs, we believe, could thus be yet another worthwhile endeavor.

### Disease-specific effects

In addition to these classical effects, we ascertained evidence of several disease-specific effects of vitamin D on disease progression or severity of HKMDs symptoms. An aggravating impact of vitamin D deficiency was described in Huntington disease and RLS. There are also, although somewhat contradictory, reports on the beneficial effects of vitamin D supplementation in RLS (Table 2). These investigations were all rather small, making it difficult to generalize the results. As seen before, supplementation for vitamin D was more widely studied in PD than in HKMDs. There are three prospective PD studies and one small meta-analysis but results, unlike as seen in RLS, were rather disappointing [54].

**Table 2:** Characteristics of vitamin D supplementation studies in patients with hyperkinetic movement disorders.

HKMDs	Author (year)	Subjects (n)	Study design	Period	Dose	Severity	Other effects

**RLS**	Wali et al. (2015) [20]	def. pts (12)	open label	12 w	28,000 IU p.o./w or 200,000 IU i.m./m	improvement	vitamin D serum level correction
	Wali et al. (2019) [21]	all pts (22)	RCT	12 w	50,000 IU p.o./w	no improvement	
	Tutuncu et al. (2020) [22]	def. pts* (19)	SCCS	8 w	50,000 IU p.o./w	improvement	vitamin D serum level increase
**Tics**	Gemawat et al. (2017) [29]	all pts (34)	RCT	24 w	n. s.	improvement	
L-dopa-induced dyskinesia**	Habibi et al. (2018) [36]	all pts (120)	RCT	12 w	1000 IU p.o./d	no improvement	

RCT: randomized controlled trial; SCCS: prospective self-controlled case study; n.s.: not specified; p.o.: per os; i.m.: intra muscular;/d: daily;/w: weekly;/m: monthly. def. pts.: Vitamin D deficient patients.

The pathophysiological process by which vitamin D deficiency might lead to the development and progression of movement disorders, is not yet fully understood. High concentrations of vitamin D metabolites and of vitamin D receptor proteins are found in the basal ganglia and connected structures [55]. There, vitamin D may function as a modulator in brain development and as a neuroprotectant [55]. One of the proposed factors is that hypovitaminosis D causes apoptosis by diminishing the expression of cytochrome C, thereby decreasing the cell cycle of neurons [56]. This, one would assume, would especially occur in areas of the brain with a high vitamin D receptor and vitamin D metabolite concentration. Another important mechanism is that vitamin D has exhibited an association with the regulation of the synthesis of nerve growth factor that is responsible for the growth and survival of neurons [57]. Finally, vitamin D can also act through several other mechanisms, including effects on protein expression, oxidative stress, inflammation, and cellular metabolism [58,59].

However, the causal link to neurodegeneration is still a matter of debate, especially with regard to the effect of vitamin supplementation on disease progression [60]. To date, as for HKMDs, real proof of clinical benefits are inconclusive [60]. This highlights that there is a strong need for randomized clinical trials examining vitamin D supplementation in patients with neurodegenerative disorders with the focus to optimize time, efficacy, and appropriate dosing [60].

### Limitations

There are several limitations of our review that warrant mention. It had to rely on few studies with large differences in sample sizes, which limited our power to detect the effects of moderators and publication bias. This lack of power may be particularly important when it comes to the effect of supplementation. Here different treatment regimens regarding dose application mode and duration of treatment were used. Also, there were differences in patient selection, as some gave the treatment to all patients and others only to those with vitamin D deficiency. Some of the trials did not specify the primary outcome. All this makes a comparison of results difficult and limits the degree to which we can make conclusions about effectiveness. In addition, it is of note that some of the authors used different definitions of vitamin D deficiency. While there was an accordance on measuring serum levels of 25-hydroxyvitamin D (25(OH)D), the cut-off levels differed among different study groups. These incongruences should be taken into account in the further investigations on HKMDs that, we believe, are earnestly needed.

Finally, one could also question the composition of our survey. HKMDs are disparate diagnoses, and one might argue that it is unclear why for this study, they should be categorized together. HKMDs are phenotypically linked by the presence of excess unwanted movements but also often by balance and gait difficulties that might make patients prone to an increased risk for falls. It is well known that osteoporosis to a large extent caused by vitamin D deficiency increases the risk of fall-related fractures [61]. It is also established that with a sufficient dose of oral vitamin D (700 to 1000 IU a day), fractures can be reduced by at least 20% [62]. If we would have sound evidence that vitamin D deficiency is substantially frequent in patients suffering from HKMDs, and that the positive effects are similar in HKMDS as in the general population, then it would be essential to provide guidelines to routinely test HKMDs patients for vitamin D levels, even those who have not yet had falls. The aim would be to prevent fractures and its serious consequences.

In addition, HKMDs share common neural pathways involved in voluntary motor control, often described as the basal ganglia network [61]. The pathophysiology of HKMDs appears to result from similar alterations in the physiological properties of the neurons in these areas [61]. Based on the commonality of pathomechanisms that underlie their development, it is not surprising that surgical therapies for HKMDs would effectively target common regions of the brain, and that pharmacological therapies would be aimed at receptors that regulate these same neural pathways [61]. Several properties of vitamin D seem to have an affinity to these pathways and to have a special effect on neurons that are particularly affected in HKMDs. This makes the investigation of the influence of vitamin D in HKMDs especially interesting not only on a clinical but also on a basic science level.

## Conclusion

While the connection between vitamin D deficiency and various aspects of HKMDs is likely to be complex and is not yet fully understood, the somewhat limited data extracted from this study still suggest that valuable lessons could be learned from investigating these mechanisms further. However, as we have shown, for HKMDs, there are still many areas where high-quality data are lacking, and in some, there are none at all. This is particularly striking when we compare the information available for HKMDs with that of PD and other neurological diseases and given the importance of vitamin D for the prevention of injuries and possibly also disease development and progression. We hope that this study may prompt HKMDs researchers to close the many gaps that we have indicated in our study. At the same time, we believe there is already sufficient data for practicing physicians to consider checking vitamin D levels in their patients with HKMDs, especially in those of advanced age or a history of falls, and to supplement where necessary.
